# Dicer-like Proteins Regulate the Growth, Conidiation, and Pathogenicity of *Colletotrichum gloeosporioides* from *Hevea brasiliensis*

**DOI:** 10.3389/fmicb.2017.02621

**Published:** 2018-01-04

**Authors:** Qiannan Wang, Bang An, Xingrong Hou, Yunfeng Guo, Hongli Luo, Chaozu He

**Affiliations:** Hainan Key Laboratory for Sustainable Utilization of Tropical Bioresources and College of Biology, Institute of Tropical Agriculture and Forestry, Hainan University, Haikou, China

**Keywords:** *Colletotrichum gloeosporioides*, dicer like proteins, conidiation, pathogenicity, proteomics analysis

## Abstract

*Colletotrichum gloeosporioides* from *Hevea brasiliensis* is the hemibiotrophic fungi which could cause anthracnose in rubber trees. Dicer like proteins (DCL) were the core enzymes for generation of small RNAs. In the present study, the knocking-out mutants of two dicer like proteins encoding genes of *C. gloeosporioides* were constructed; and functions of two proteins were investigated. The results showed that DCL play important roles in regulating the growth, conidiation and pathogenicity of *C. gloeosporioides*; and there is a functional redundancy between DCL1 and DCL2. Microscopy analysis and DAB staining revealed that loss of penetration ability into the host cells, instead of the decreased growth rate, was the main cause for the impaired pathogenicity of the ΔDcl1ΔDcl2 double mutant. Proteomics analysis suggested that DCL proteins affected the expression of functional proteins to regulating multiple biological processes of *C. gloeosporioides*. These data lead to a better understanding of the functions of DCL proteins in regulating the development and pathogenesis of *C. gloeosporioides*.

## Introduction

Rubber tree (*Hevea brasiliensis*) is one of important tropic economic crops which serve the primary resource of nature rubber. The anthracnose of rubber tree caused by *Colletotrichum* led to serious economic losses in Hainan province, the main rubber planting area of China. In the preliminary work, we found that *Colletotrichum gloeosporioides* is the main pathogen of anthracnose. To explore the molecular mechanism of its pathogenicity, the genome of *C. gloeosporioides* from was sequenced (data unpublished). *Colletotrichum* species are common plant pathogens which have a hemibiotrophic lifestyle, meaning that they are biotrophic at initial stage of infection to their host cells and necrotrophic at later phase (Koeck et al., 2011; O'Connell et al., 2012). It is vital for pathogens to overcome the plant immune system at the biotrophic stage. Effector proteins were thought to play as molecular weapons to suppress the plant immunity (Jones and Dangl, 2006; Göhre and Robatzek, 2008; Rafiqi et al., 2012). When interact with the host plant, *Colletotrichum higginsianum* could secret a lot of effector proteins by appressoria and intracellular hyphae to manipulate the plant physical processes to ensure its successful evasion (Stephenson et al., 2000; Kleemann et al., 2012; Pumplin and Voinnet, 2013).

Small RNAs, about 20–30 nucleotide long, are small non-coding RNAs found in living cells. Small RNAs could regulate the gene expression at both posttranscriptional and transcriptional levels, which is known as gene silencing (PTGS) (Matzke and Matzke, 1995), quelling (Romano and Macino, 1992), and RNA interference (RNAi) (Bass, 2000). There are three major classes of small RNAs identified in eukaryotes by now: small interfering RNA (siRNA), microRNA (miRNA) and piwi-interacting RNA (piRNA) (Carthew and Sontheimer, 2009; Moazed, 2009), and each class of small RNAs has diverse functions. In animals and plants, small RNAs are proved to play vital roles in regulating multiple biological processes, including morphogenesis, hormone signaling and stress responses (Bartel, 2004; Rubio-Somoza et al., 2009; Mendell and Olson, 2012). In fungi, small RNAs also play multiple biological functions, including regulation of heterochromatin formation in *Schizosaccharomyces pombe* (Volpe et al., 2002), controlling of transposon in *Neurospora crassa* (Nolan et al., 2005), and mediating defense against virus in *Aspergillus nidulans* (Ding and Lu, 2011). Recent works show that the fungal pathogens could also manipulate the immunity system of the host plant by generating small RNAs. Weiberg et al. (2013) found that *Botrytis cinerea* (a necrotrophic pathogen) could secret small RNAs into the host plant, hijack the host Argonaute (AGO) proteins to decrease the plant immunity. Dicer like proteins (DCL) are important for generation of small RNAs in living cells (MacRae et al., 2006; Xue et al., 2012). Many animals and fungi have two Dicer-like genes (*Dcl*), and plant have four or six Dicer-like genes. In the human endothelial cells, microRNAs regulate the gene expression in a dicer dependent manner (Suárez et al., 2007). In *Drosophila* cells, DCL1 and DCL2 play distinct roles in processing miRNA precursors and siRNA precursors (Lee et al., 2004). In *Arabidopsis thaliana*, DCLs play multiple roles in regulating biological processes, such as flowering process (Schmitz et al., 2007) and virus induced host silencing (Blevins et al., 2006). In fungi, DCLs of *B. cinerea* are proved to be involved in vegetative growth and processing of small RNAs (Weiberg et al., 2013). The functions of DCLs in *C. gloeosporioides* are still unclear. Therefore, in the present study, two *Dcl* genes in *C. gloeosporioides* were knocked out and their functions in regulating growth, conidiation and pathogenicity were investigated.

## Materials and methods

### Fungal strains and culture conditions

*Colletotrichum gloeosporioides* was isolated from the *H. brasiliensis* with Anthracnose; furthermore, the genome of the *C. gloeosporioides* was sequenced for exploration of possible mechanism of pathogenicity (data unpublished). The *C. gloeosporioides* was used as recipient strain for the transformation experiments and as a WT control. All the *C. gloeosporioides* strains were grown on potato dextrose agar (PDA) at 28°C.

### Vector constructions

**Figure 1:**
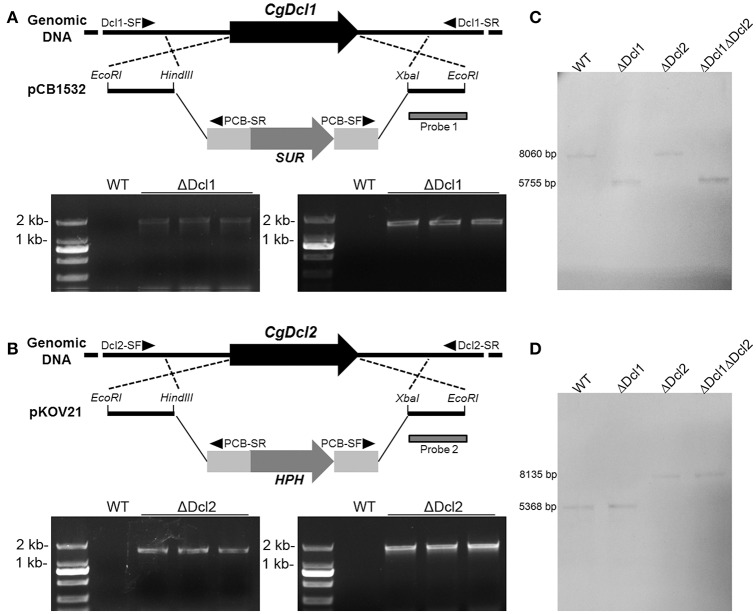
The gene deletion strategy and PCR confirmation of *Dcl1* **(A)** and *Dcl2* **(B)** in *C. gloeosporioides*. Putative mutants were screened with diagnostic primers, which were indicated with black triangles. **(C,D)** Confirmation of the correct recombination of the deletion cassettes to the gene loci with Southern blot. The downstream flanking of the sequences were used as the probes for Southern blot analyses.

Vector pCB1532 carrying the acetolactate synthase gene (SUR) cassette from *M. oryzae* (Yang et al., 2013) conferred resistance to chlorimuron ethyl (a sulfonylurea herbicide) and pKOV21 carrying the hygromycin phosphotransferase gene (HPH) conferred resistance to hygromycin were used to construct the replacement vectors. The replacement vector for *Dcl1* was designed as described in Figure 1A: the 5′ flanking region and 3′ region of the *Dcl1* nucleotide were amplified by use of the primer pairs 1/2 and 3/4, respectively; then the two fragments were excised with EcoRI/HindIII and XbaI/EcoRI respectively and ligated into vector pCB1532 to construct the replacement vector. The replacement vector for *Dcl2* was designed as described in Figure 1B: the 5′ flanking region and 3′ region of the *Dcl2* nucleotide were amplified by use of the primer pairs 5/6 and 7/8, respectively; then the two fragments were excised with EcoRI/HindIII and XbaI/EcoRI respectively and ligated into vector pKOV21 to construct the replacement vector. The two deletion vectors were excised with EcoRI before transformation of the *C. gloeosporioides* strain. Vector pBS-NEO carrying the Neomycin phosphotransferase gene (NPTII) conferred resistance to Geneticin (G418) was used to construct the complementation vector. To get the complementation vector, a 6.4 kb fragment containing *Dcl1* the sequence together with a 1.4 kb upstream nucleotide and a 5.9 kb fragment containing *Dcl2* the sequence together with a 1.3 kb upstream nucleotide were amplified by using the primers 21/22 and 23/24; then the two fragments were excised with XbaI/BamHI and SacII/XbaI respectively and ligated into the vector pBS-NEO (Figure S2A). The complementation vector was excised with XbaI before transformation of the double deletion mutant strain.

### Transformation of *C. gloeosporioides*, PCR diagnosis, and single conidia purification

Conidia was inoculated into the 200 mL potato broth to make the initial number of conidia to 10^5^ conidia ml^−1^ and then cultured at 28°C, 150 rpm for 24 h. Then the mycelium were collected with the nylon membrane, washed two times with 1 M sorbitol and transfer to the 1 M sorbitol containing the 10 mg ml^−1^ lysing enzyme (Sigma-Aldrich). Then the mycelium was incubated at 28°C, 100 rpm for 3 h to catalyze the cell wall. After that, the protoplast were filtered with nylon membrane and collected by centrifugation with 2,000 rpm at 4°C, washed two times and resuspened with STC buffer (1 M sorbitol, 50 mMTris-Cl, 10 mM CaCl_2_, pH 7.4) to the final concentration of 10^8^ ml^−1^ CFU. For the transformation, 100 μL linearized replacement vector was added into the 200 μL protoplast and the mixture was incubated on ice for 20 min. Then 1 ml 40% PEG dissolved in STC buffer was added into the protoplast mixture and placed for 20 min at 28°C. After the transformation, 5 mL liquid regeneration medium (1 g L^−1^ yeast extract, 1 g L^−1^ casein, 6 M sucrose) was added into the protoplast and cultured at 28°C, 100 rpm for 4 h. Then the regenerated protoplast was transfer into the regeneration medium with 1% agar at about 50°C, mixed gently and spread on the petri dish. After the agar concreted, same volume of regeneration medium with 1% agar containing 100 μg ml^−1^ chlorimuron ethyl or 200 μg ml^−1^ hygromycin was spread on the upper level to select the transformants. For generation of the double mutants, the *Dcl1* deletion mutants were used as the recipient strain to conduct the protoplast preparation and transformation to delete *Dcl2*. The chlorimuron ethyl-resistant or the hygromycin-resistant strains were isolated and analyzed by PCR with the primer pairs as showed in Figure 1, which are diagnostic for homologous integration of 5′ part and 3′ part. Then the correct transformants were purified by single conidia isolations. Single conidial isolates were obtained by spreading 100 μL of conidial suspension (10^4^ conidia mL^−1^) on Malt extract agar medium (BD, USA) plates containing 100 μg mL^−1^ chlorimuron ethyl or 300 μg mL^−1^ hygromycin. For generation of the complementation mutants, the *Dcl1* and *Dcl2* double deletion mutants were used as the recipient strain to conduct the transformation. Regeneration medium containing 200 μg ml^−1^ G418 was used to select the transformants. PCR with the primer pairs 21/22 and 23/24 were used for integration diagnosis. After that, Single conidial isolation was conducted to purify the transformants.

### Southern blot analysis

The genomic DNA of the WT and the mutants were extracted and excised with EcoRI. The DNA probes was amplified and labeled by digoxin using digoxigenin-dUTP (Roche) as shown in Figure 1. The DNA band with the hybridized probe was visualized using an enzyme immunoassay and enzyme catalyzed color reaction with NBT/BCIP (Roche).

### Growth and conidiation assay

Wild-type and the mutant strains were grown for 3 day on PDA medium and a disk of hypha with diameter of 1 mm was removed from the growing edge; then the disk of hypha was inoculated on the complete medium (CM) and minimal medium (MM). After culture for 5 days, the diameter was recorded and the growth rate was calculated. Conidia were harvested from *C. gloeosporioides* strain grown on PDA medium for 12 day, inoculated into 50 mL liquid CM medium to the final concentration of 10^3^ mL^−1^, and cultured at 28°C, 150 rpm for desired time. Then the conidia number after incubation for 3 and 4 days were numbered under microscope.

### Pathogenicity assay

For the pathogenicity assay, conidia of the WT and the mutants were collected, washed two times with ddH_2_O and resuspended in a solution of 5% Sabouraud Maltose Broth (Difco) to a final concentration of 2 × 10^5^ conidia mL^−1^. Droplets (5 μL) of the conidial suspensions were used to inoculate the detached “light green” leaves from rubber tree Varity 73-3-97. The inoculated leaves were kept in a moist chamber at 28°C under natural illumination for 4 days and the disease symptoms were scored. Each treatment contained three replicates of 15 leaves and the entire experiment was repeated three times.

### Penetration ability assay

Conidia was prepared as mentioned above and resuspended with sterilized ddH_2_O to the final concentration of 2 × 10^5^ conidia mL^−1^. Onion epidermis was harvested and put on water agar plates. Then the onion epidermis was inoculated with 20 μL of conidia suspension and kept in a moist chamber at 28°C for desired time. After that, the infection structures were analyzed with microscope. The penetration rates of the conidia were quantified after inoculation for 12 h. About 50 conidia were counted under the microscope, and each treatment contained three replication. Sterilized cellophane paper were put on malt extract agar medium, inoculated with 10 μL of conidial suspension, and kept in a moist chamber at 28°C for desired time. The germination were observed by using microscope.

### Oxidative burst assay

The rubber tree leaves were wounded with sterilized needle. Conidia of WT and mutant strains was prepared as mentioned above, resuspended with 5% Sabouraud Maltose Broth (Difco) to a final concentration of 2 × 10^5^ conidia mL^−1^. Droplets (5 μL) of the conidial suspensions were used to inoculate the wounded leaves. The inoculated leaves were kept in a moist chamber at 28°C under natural illumination for 24 h. Then the oxidative burst was analyzed by staining the leaves with DAB (Sigma-Aldrich) according to the protocol from Daudi et al. (2012). Wounded leaves inoculated with the only 5% Sabouraud Maltose Broth were used as control check (CK).

### Proteomic analysis

Protein extraction, two-dimensional (2D) gel electrophoresis, image analysis and in-gel digestion were carried out according to An et al. (2016). Wild-type and the mutant strains were grown for 3 day on cellophane paper plated on the Malt extract agar medium. The hyphae were collected and disrupted in liquid nitrogen by grinding in a mortar with a pestle. Total protein were extracted with the extraction buffer containing 0.5 M Tris-HCl, pH 8.3, 2% (v/v) NP-40, 20 mM MgCl_2_, 2% (v/v) β-mercaptoethanol, and 1 mM PMSF. After removing the cell debris by centrifugation, the supernatant was extracted with an equal volume of Tris-HCl (pH 7.8) buffered phenol. After centrifugation, proteins were precipitated from the final phenol phase with 5 vol of ice-cold saturated ammonium acetate in methanol overnight at −20°C. Then the proteins were collected by centrifugation and washed twice with cold saturated ammonium acetate in methanol and acetone. The precipitate was air-dried for 1 h at 4°C and then solubilized in the thiourea/urea lysis buffer containing 2 M thiourea, 7 M urea, 4% (w/v) CHAPS, 1% (w/v) DTT, and 2% (v/v) carrier ampholytes of pH 3–10. For two-dimensional (2D) gel electrophoresis, aliquots of 650 μg of proteins resolved in 340 μL of sample buffer (7 M urea, 2 M thiourea, 4% (w/v) CHAPS, 1% (w/v) DTT, 2% (v/v) carrier ampholytes (pH 3-10), and 0.001% (w/v) bromphenol blue) were used to rehydrate gel strips (Immobiline DryStrip pH 4–7, 18 cm; GE Healthcare) for 16 h. The first-dimensional IEF, equilibration of the IPG strips and the second-dimensional separation were conducted with the Ettan IPGphor III and the Ettan DALTsix (GE Healthcare) systems according to the manufacturer's instructions. Protein spots whose expression levels changed by >2-fold were excised for protein identification using a MALDI-TOF/TOF mass spectrometer (Ultrafle Xtreme; Bruker Daltonics, Billerica, MA, USA). MS data were uploaded to Mascot for database searching on the Matrix Science (London, UK) public website (http://www.matrixscience.com) and searched against the NCBInr protein database with BIOTOOLS software (v.3.2; Bruker Daltonics). Search parameters were set as taxonomy: Fungi; enzyme: trypsin; max missed cleavages: 1; fixed modifications: carbamidomethyl (C); variable modifications: oxidation (M); peptide mass tolerance: ±100 ppm; fragment mass tolerance: ±0.5 Da. In addition, a peptide charge of 1 + and a monoisotopic mass were selected and the instrument type was set to MALDI-TOF-TOF.

### Quantitative RT-PCR analysis

Wild-type and the mutant strains were grown for 3 day on cellophane paper plated on the Malt extract agar medium. The hyphae were collected and disrupted in liquid nitrogen by grinding in a mortar with a pestle, then the RNA was extracted using TRIzol Reagent (Invitrogen). Reverse transcription was conducted with RevertAid RT Reverse Transcription Kit (Thermo Fisher) according to the manufacturer's instructions. Quantitative RT-PCR analysis was performed with the LightCycler 96 System (Roche). The beta-tubulin-1 gene was used as an endogenous control for normalization. Relative expression levels were estimated using the 2^−ΔΔCt^ method. The primers used for quantitative RT-PCR are listed in Table S1.

## Results

### Generation of the ΔDcl1, ΔDcl2, ΔDcl1ΔDcl2, and the complementation mutants

Two dicer like protein (DCL) coding gene were identified in *C. gloeosporioides* using the genome database. The *Dcl1* consists of 4,900 bp and codes a protein composed of 1564 amino acids; the *Dcl2* consists of 4618 bp and codes a protein composed of 1453 amino acids. To explore their functions in *C. gloeosporioides*, the nucleotides of two genes were deleted by a replacement strategy as shown in Figure 1. The Chlorimuron ethyl or Hygromycin resistant colonies were analyzed for homologous integration by PCR. To verify the integration locus by PCR, primer pairs with one primer being located outside and one inside the construct were used. As shown in Figure 1, at least three mutants showed the both diagnostic fragments of 5′ and 3′ flanking region. The fragments were sequenced to ensure flawlessness. The results showed that the replacement fragments were correctly integrated into the *Dcl* loci. The knocking-out mutants were named ΔDcl1 and ΔDcl2 respectively. The results of PCR diagnosis of the double mutant (ΔDcl1ΔDcl2) showed all the diagnostic fragments. All the transformants were purified by single conidia isolation. The detection of WT nuclei of single conidia isolations was implemented by PCR with gene primer pair 17/18 and 19/20. The Southern blot assay indicated that the ΔDcl1, ΔDcl2, and the ΔDcl1ΔDcl2 mutants all showed single homologous integration; and the hybrid bands were consistent with expected DNA sequence lengths (Figures 1C,D). Since the purified single conidia isolates of the three mutants showed the identical phenotypes both in growth rate and pathogenicity, only one strain of each kind of mutant was chosen for detailed studies. Complemented mutant strains were diagnosed by PCR with the primer pairs 21/22 and 23/24. The correct transformants were also purified by single conidia isolation.

### DCLs are involved in vegetative growth and conidiation

**Figure 2:**
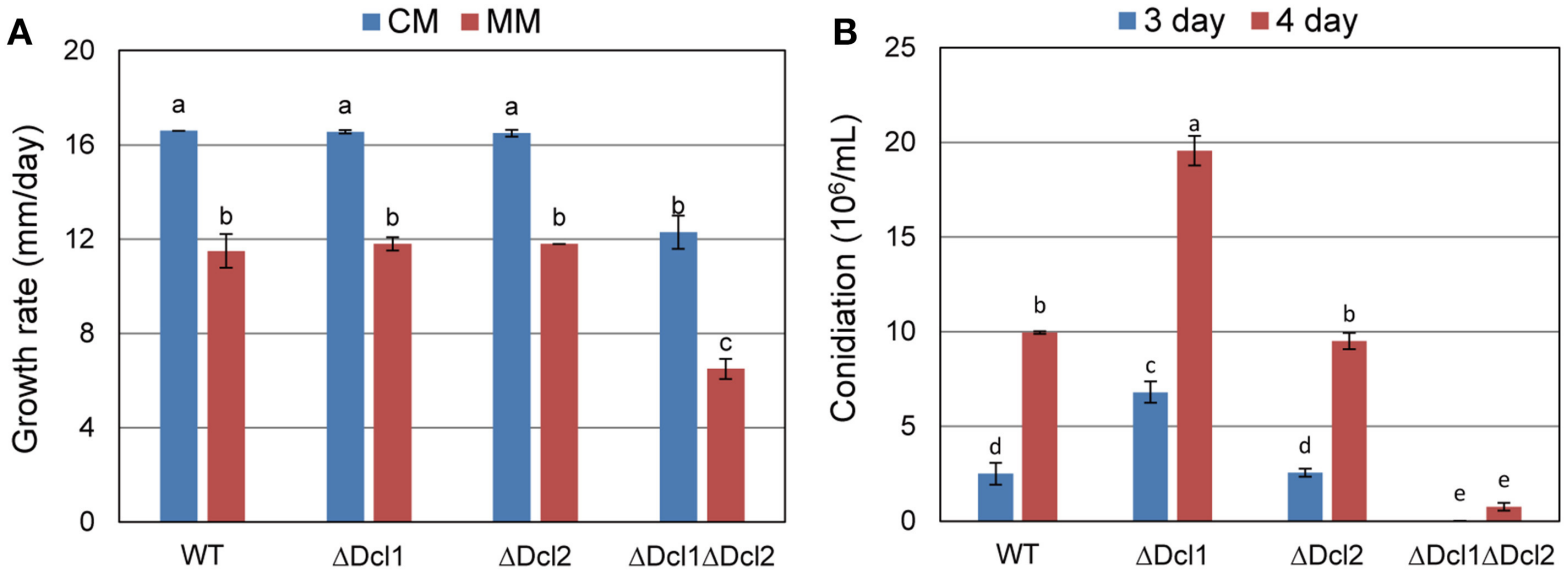
Growth rate and conidiation of *C. gloeosporioides*. **(A)** Growth rate of WT and the mutant strains on complete medium (CM) and minimal medium (MM) for 5 days. **(B)** Conidation of WT and the mutant strains. Bars represent standard deviations (SD). Columns with different letters indicate significant difference (*P* < 0.05).

The ΔDcl1 and ΔDcl2 showed similar growth rate to WT when cultured on complete medium or minimal medium; while the vegetative growth of ΔDcl1ΔDcl2 was obviously decreased compared with WT (Figure 2A). When cultured in liquid medium, ΔDcl2 generated similar amount of conidia compared with WT, while ΔDcl1 showed an obvious increase in conidiation; for the ΔDcl1ΔDcl2, the conidiation was seriously impaired (Figure 2B). After reintroducing the *Dcl1* and *Dcl2* nucleotides back into the double deletion mutant, the growth rate of the complemented mutant strain was restored (Figure S2B).

### DCLs are required for pathogenicity and penetration process

**Figure 3:**
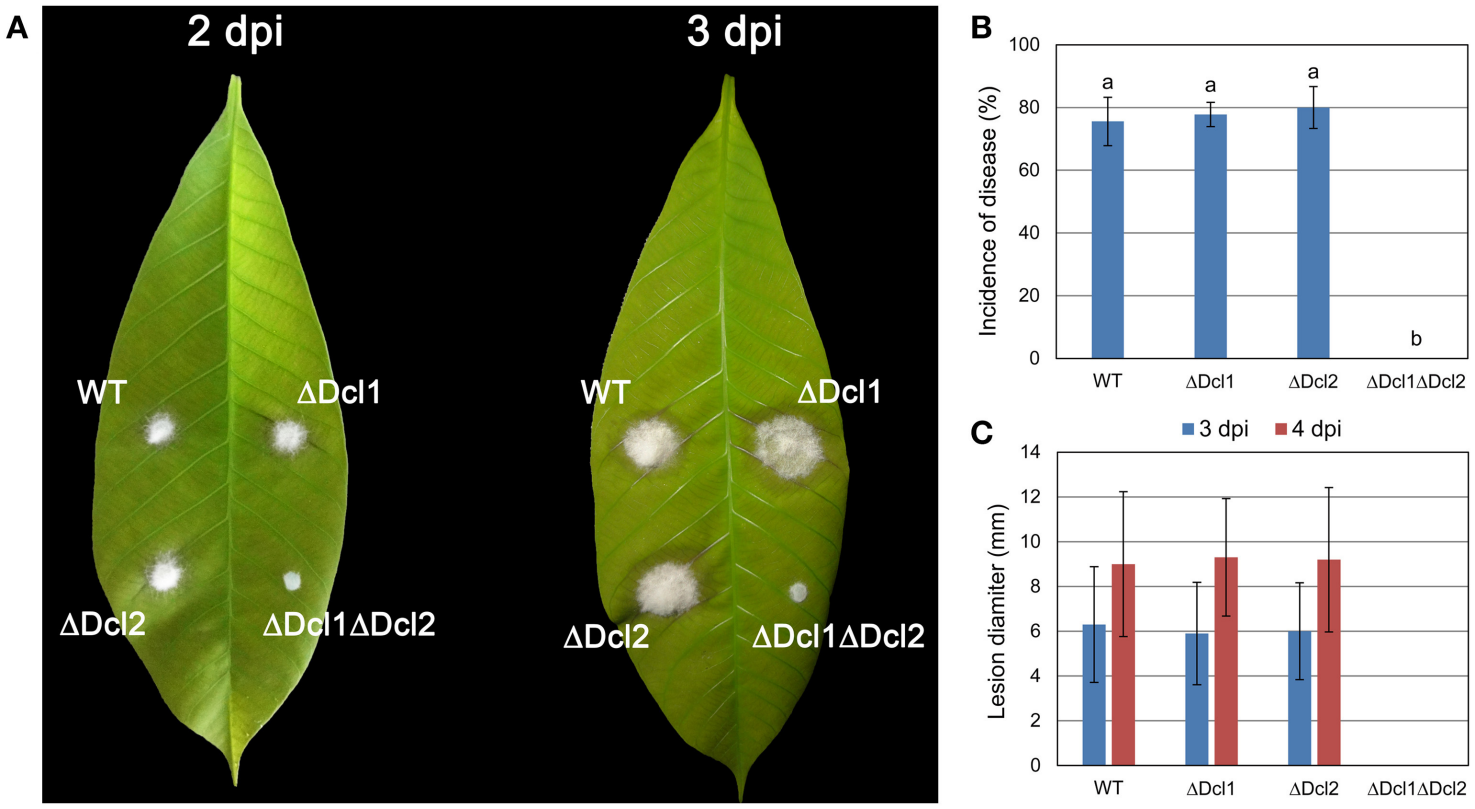
Virulence assay on rubber tree leaves. **(A)** Disease symptoms of rubber tree leaves at 2 day post inoculation (dpi) and 3 dpi. **(B)** Mean incidence of disease of rubber tree leaves at 3 dpi. **(C)** Mean lesion diameters after 2 and 3 dpi. Bars represent standard deviations (SD). Columns with different letters indicate significant difference (*P* < 0.05).

Detached “light green” leaves from rubber tree Varity 73-3-97 were used to determine the pathogenicity of the mutant strains. As shown in Figure 3, about 80% conidia of the WT, ΔDcl1 and ΔDcl2 strains was able to invade the leaves and cause disease, and the lesion diameter were about 6 mm and 9 mm at 2 and 3 dpi. But ΔDcl1ΔDcl2 completely lost the pathogenicity on the rubber leaves. The lesions produced by the complemented mutant strain were nearly similar to those produced by the WT strain (Figure S2C).

**Figure 4:**
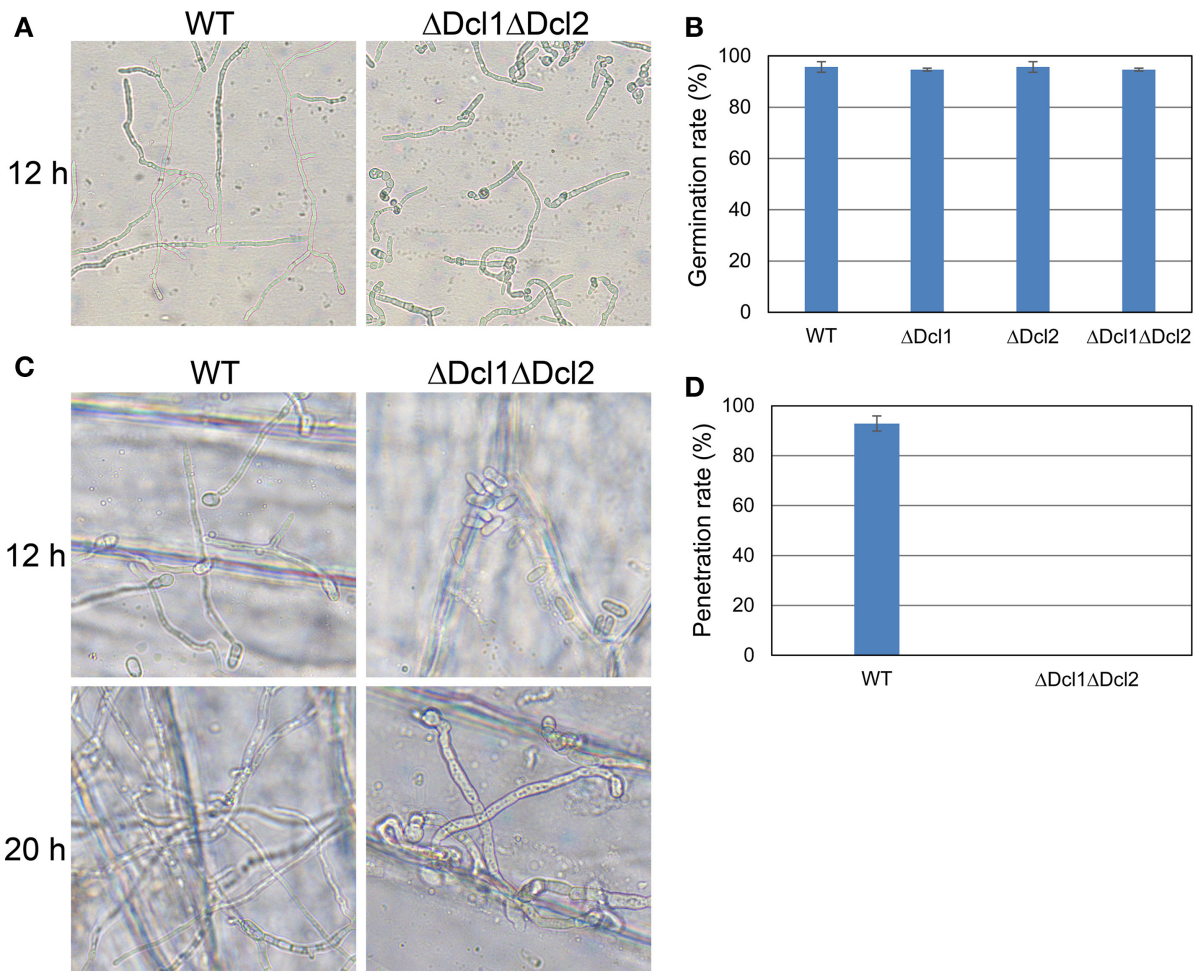
Growth assays of WT and ΔDcl1ΔDcl2 mutant on cellophane and onion epidermis. **(A)** Germination behavior after culture for 12 h. **(B)** Germination rate of conidia of all the strains after culture for 6 h. Bars represent standard deviations (SD). **(C)** Penetration assay of WT and ΔDcl1ΔDcl2 mutant on onion epidermis after inoculation for 12 and 20 h. **(D)** Penetration rate of conidia of WT and ΔDcl1ΔDcl2 inoculated on onion epidermis for 12 h.

**Figure 5:**
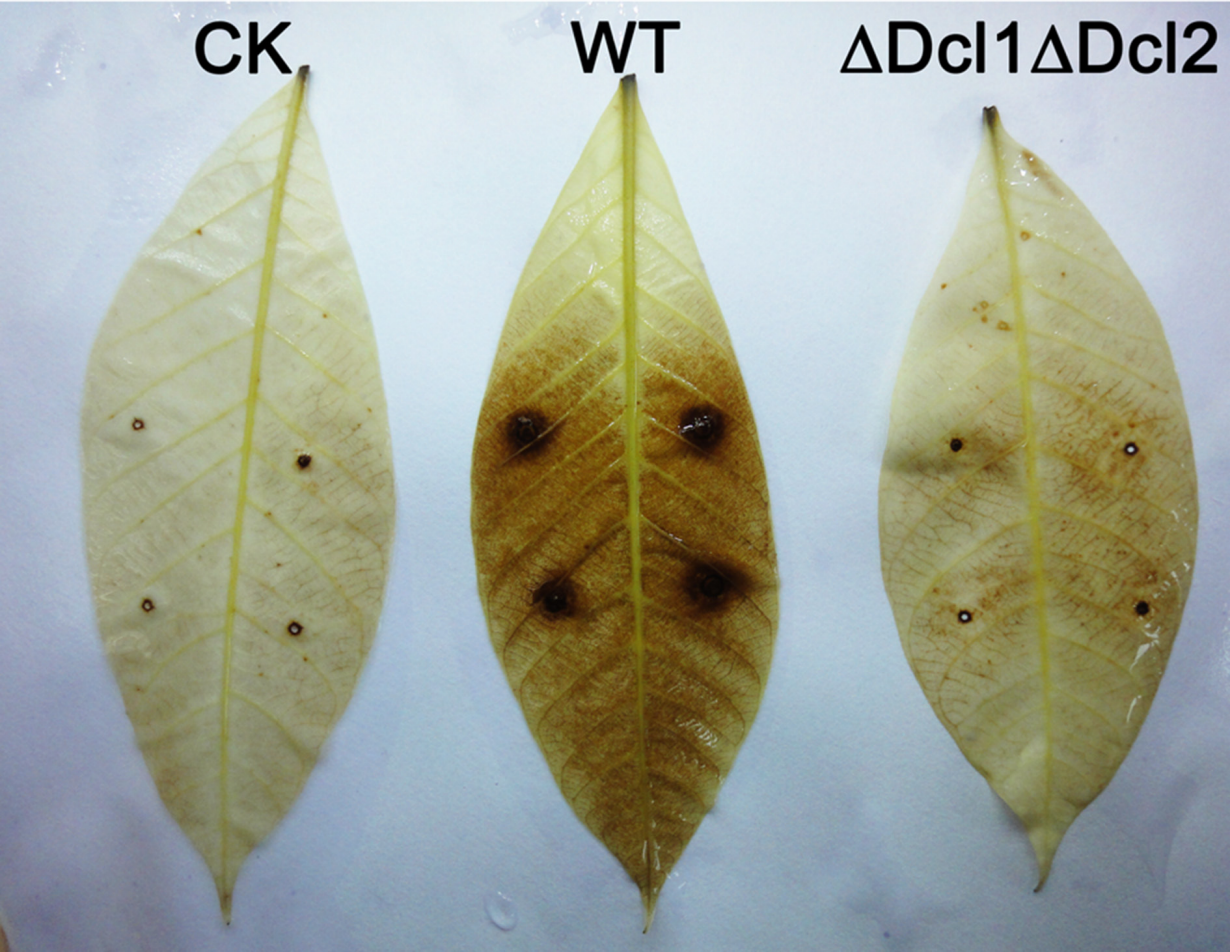
Oxidative burst assay of rubber tree leaves inoculated with WT and ΔDcl1ΔDcl2 mutant by DAB staining.

To gain a further insight, we tested the conidial germination and penetration on onion epidermis and cellophane paper. After incubation of 12 h, all the conidia of WT germinated and penetrated into the onion epidermis; but the conidia of ΔDcl1ΔDcl2 mutant did not geminated at all; after 24 h, mycelium of WT formed complex networks in onion cells; but the conidia of ΔDcl1ΔDcl2 mutant had just germinated with abnormal germ tubes (Figure 4A). When cultured on the cellophane paper, conidia of ΔDcl1ΔDcl2 mutant germinated at the same rate as WT, although the growth rate was decreased (Figures 4B,C). These results suggested that the penetration ability of ΔDcl1ΔDcl2 mutant was impaired. DAB staining showed that when the wounds were inoculated with the conidia of WT, the rubber leaves could generate significant oxidative burst compared with the CK (inoculated with only Maltose Broth); but that inoculated with ΔDcl1ΔDcl2 mutant did not show significant increase in peroxide (Figure 5).

### DCLs are required for generation of functional proteins

**Figure 6:**
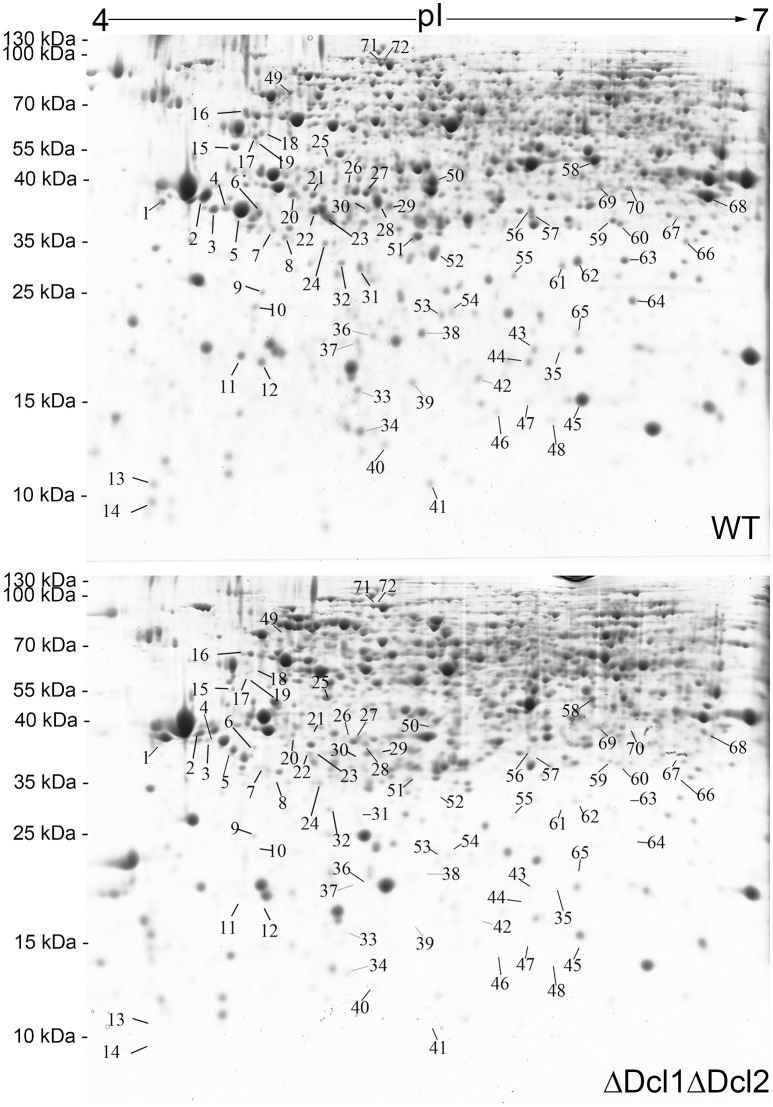
Two-dimensional patterns of proteins of WT and ΔDcl1ΔDcl2 mutant of *C. gloeosporioides* from *H. brasiliensis*. Arrows indicate protein spots which down regulated in abundance more than 2-fold between WT and the mutant. The protein spots are numbered corresponding to those in Table 1.

A comparative proteomic analysis was conducted to identify the proteins that regulated by DCLs. The 2D gel electrophoresis was conducted to separate the proteins isolated from the WT and the ΔDcl1ΔDcl2 mutant; approximately 1680 protein spots were detected on CBB-stained 2D gels. The quantitative image analysis revealed a total of 72 protein spots that showed at least 2-fold down regulation in abundance (P < 0.05) in the double mutant (Figure 6). Besides, only a few protein spots showed up-regulated with abundance change smaller than 2 fold in the mutant. So only the down-regulated protein spots were further analyzed. The 72 down-regulated protein spots were excised and submitted to tandem mass spectrometry and identified by database searching with the Mascot search engine (Table 1). The identified proteins were classified into 9 functional categories based on the FunCatannotation scheme (http://ibis.helmholtz-muenchen.de/funcatDB/), including protein synthesis, cell cycle, chemical metabolism, hydrolytic enzyme, signal transduction, transport, charpones, cell structure and unknown.

**Table 1:** Proteins Identified in *C. gloeosporioides* hyphae by quadrupole time-of-Flight tandem mass spectrometry.

**Spot**	**Protein name**	**Accession number**	**Theo. *M*_r_ (kDa)**	**pI**	**NP**	**SC (%)**	**WT vs. double mutant**
**CELL CYCLE**							
20	DNA damage checkpoint protein rad24	ELA23670.1	29.81	4.54	15	44	3.25
21	DNA damage checkpoint protein rad24	ELA23670.1	29.81	4.54	9	30	5.75
22	DNA damage checkpoint protein rad24	ELA23670.1	29.81	4.54	15	46	9.64
23	DNA damage checkpoint protein rad24	ELA23670.1	29.81	4.54	13	43	2.15
71	Cell division control protein cdc48	ELA32075.1	92.79	4.69	25	28	∞
72	Cell division control protein cdc48	ELA32075.1	92.79	4.69	20	24	∞
**PROTEIN SYNTHESIS**							
9	Eukaryotic translation initiation factor 1a	ELA28354.1	17.97	4.63	6	36	2.17
29	Eukaryotic translation initiation factor 3 subunit I	ELA35605.1	38.15	5.64	11	33	15.82
61	Elongation factor 1-gamma	ELA38399.1	46.72	6.4	8	16	6.76
62	Elongation factor 1-gamma	ELA38399.1	46.72	6.4	11	18	6.88
28	40s ribosomal protein s0	ELA38230.1	28.86	5.37	11	34	2.77
66	40s ribosomal protein s0	ELA38230.1	28.86	5.37	5	15	∞
33	Rnp domain-containing protein	ELA28088.1	43.08	7.08	2	6	14.97
38	ATP-dependent RNA helicase eif4a	ELA31107.1	44.92	4.78	6	14	14.05
56	60s acidic ribosomal protein p0	ELA31112.1	36.55	4.46	9	24	4.73
57	60s acidic ribosomal protein p0	ELA31112.1	36.55	4.46	9	24	∞
64	Peptidyl-prolyl cis-trans isomerase	ELA35461.1	40.30	5.37	8	21	8.93
**CHAPERONES**							
1	Heat shock protein 90	ELA23732.1	79.45	4.75	10	13	∞
2	Heat shock protein 90	ELA23732.1	79.45	4.75	18	18	3.01
3	Heat shock protein 90	ELA23732.1	79.45	4.75	17	17	6.33
4	Heat shock protein 90	ELA23732.1	79.45	4.75	10	13	∞
31	Heat shock protein 90	ELA23732.1	79.45	4.75	12	16	∞
5	Heat shock 70 kDa protein	ELA32774.1	70.81	4.84	19	20	8.42
6	Heat shock 70 kDa protein	ELA32774.1	70.81	4.84	13	16	4.63
7	Heat shock 70 kDa protein	ELA32774.1	70.81	4.84	10	14	∞
8	Heat shock 70 kDa protein	ELA32774.1	70.81	4.84	12	16	∞
10	Cs domain-containing protein	ELA32913.1	22.28	4.14	6	20	5.60
43	Aha1 domain family	ELA34267.1	36.29	5.28	6	19	5.04
49	Calnexin	ELA29565.1	63.15	4.76	15	23	3.83
67	Glutathione s-transferase	ELA24003.1	29.32	6.97	8	29	∞
**TRANSPORT**							
24	Rab GTPase vps21	ELA25539.1	25.33	5	9	33	2.82
25	Protein disulfide-isomerase	ELA28064.1	56.00	4.57	14	23	3.01
32	Ras small monomeric GTPase	ELA24310.1	24.37	4.63	3	10	∞
34	Alpha-mannosidase	ELA31115.1	130.90	6.4	5	4	3.64
36	Rab small monomeric gtpase	ELA36871.1	23.21	4.61	4	18	∞
37	Mitochondrial import receptor subunit tom-20	ELA36825.1	19.30	4.76	2	11	∞
41	ADP-ribosylation factor	ELA23700.1	20.98	6.8	3	18	∞
42	Clathrin light chain	ELA30885.1	26.12	4.21	6	18	∞
53	Ras GTPase	ELA23977.1	23.36	5.7	10	47	2.85
54	Ras GTPase	ELA23977.1	23.36	5.7	9	43	2.48
**CELL STRUCTURE**							
11	Actin	ELA34037.1	41.65	5.15	2	5	17.56
12	Actin lateral binding protein	ELA36900.1	18.66	4.53	12	60	∞
13	Actin lateral binding protein	ELA36900.1	18.66	4.53	8	48	∞
70	Actin-related protein 2 3 complex subunit	ELA32869.1	40.03	6.8	9	23	2.64
**HYDROLYTIC ENZYME**							
14	Aspergillopepsin-2 heavy chain	ELA36062.1	9.07	4.82	2	18	∞
15	Endochitinase	ELA36416.1	44.79	4.57	10	24	2.46
16	Aspartic endopeptidase	ELA37088.1	50.57	4.68	12	24	8.65
27	Proteasome endopeptidase complex	ELA37155.1	39.16	4.11	14	33	1.99
**SIGNAL TRANSDUCTION**							
17	Protein phosphatase	ELA27101.1	49.29	4.41	14	30	6.25
18	Protein phosphatase	ELA27101.1	49.29	4.41	9	17	6.23
19	Protein phosphatase	ELA27101.1	49.29	4.41	9	19	2.02
26	cAMP-dependent protein kinase regulatory subunit	ELA30315.1	42.08	4.59	8	19	∞
30	Protein phosphatase pp2a regulatory subunit a	ELA34582.1	69.65	4.56	11	12	7.92
35	Rho protein gdp dissociation inhibitor containing protein	ELA26991.1	33.50	6.18	8	27	∞
44	Rho protein gdp dissociation inhibitor containing protein	ELA26991.1	33.50	6.18	7	22	∞
39	Dual specificity catalytic domain containing protein	ELA30352.1	45.31	6.52	7	13	3.83
**CHEMICAL METABOLISM**							
40	Ethyl tert-butyl ether degradation	ELA35540.1	12.00	5.01	7	70	2.93
50	Aldehyde dehydrogenase	ELA36993.1	49.96	6.25	13	30	9.06
51	Aldehyde dehydrogenase	ELA36993.1	49.96	6.25	13	39	4.33
52	Aldehyde dehydrogenase	ELA36993.1	49.96	6.25	6	16	∞
68	Malate dehydrogenase	ELA31304.1	34.33	6.98	9	32	6.61
58	Aminoglycoside phosphotransferase	ELA31403.1	41.71	6.19	21	59	2.88
69	Dienelactone hydrolase family protein	ELA32769.1	30.97	6.12	5	15	2.47
60	Short chain dehydrogenase	ELA24821.1	35.86	8.11	9	26	4.02
63	Superoxide dismutase	ELA31430.1	23.00	7.04	4	21	∞
65	3-hydroxyanthranilate 3,4-dioxygenase	ELA32139.1	17.72	5.58	3	22	∞
45	CipC-like antibiotic response protein	ELA27578.1	13.82	6.09	10	63	3.80
46	CipC-like antibiotic response protein	ELA27578.1	13.82	6.09	3	24	∞
47	CipC-like antibiotic response protein	ELA27578.1	13.82	6.09	5	43	∞
59	Aminoglycoside phosphotransferase	ELA31403.1	41.71	6.19	16	52	4.21
**UNKNOW**							
48	Uncharacterized protein	ELA37769.1	12.73	5.91	4	22	∞
55	Minor allergen alt a 7	ELA26079.1	26.48	6.03	4	13	2.01

*Theo. Mr and pI: theoretical molecular mass and isoelectric point based on amino acid sequence of the identified protein. NP: the number of matched peptides. SC: amino acid sequence coverage for the identified proteins. WT vs. double mutant: average fold change of relative abundance of specific spot of WT vs. ΔDcl1ΔDcl2 mutant from three biological repeats. ∞: corresponding spot appeared in the WT but not in the ΔDcl1ΔDcl2 mutant*.

### DCLs affect gene transcript levels

**Figure 7:**
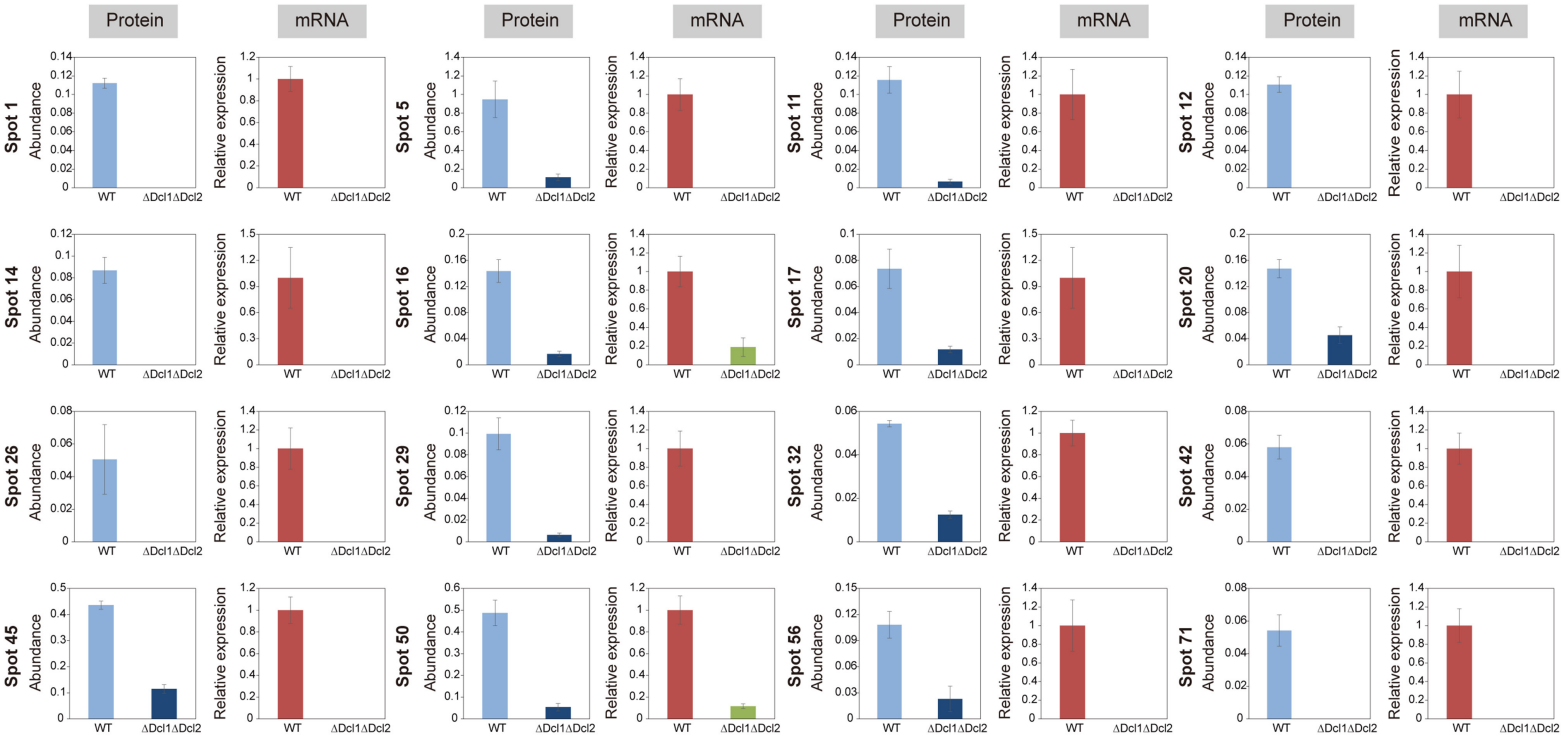
Comparison of expression profiles at the protein and mRNA levels of WT and ΔDcl1ΔDcl2 mutant of *C. gloeosporioides* from *H. brasiliensis*. The protein abundance was accessed by the protein spot volume based on the two-dimensional proteome analysis. Transcript abundance was evaluated by quantitative RT-PCR. The gene transcript levels are normalized against the beta-tubulin 1 gene, followed by normalization against the expression in WT. Bars represent standard deviations (SD).

To determine whether DCLs regulated the proteins abundance by affecting the transcript levels, the quantitative RT-PCR analysis was conducted. Our results showed that the relative levels of all of the 16 selected genes were significantly decreased; and the results was consistent with that of 2D analysis (Figure 7). These data suggested that the DCLs could directly function by affecting the transcript levels.

## Discussion

Alignment of amino acid sequences showed that the sequence of DCL of *C. gloeosporioides* has a high identity with that of *C. higginsianum, N. crassa*, and *B. cinerea*, indicating that the DCLs are well conserved in filamentous fungi (Figure S1). In the present study, knocking out of *Dcl1* or *Dcl2* did not affect the growth rate of *C. gloeosporioides in vitro*; but the double deletion mutant ΔDcl1ΔDcl2 showed an obvious reduction of growth rate. Conidiation is important for the reproduction and pathogenicity of fungi. Deletion of *Dcl2* did not influence the conidiation process; while deletion of *Dcl1* greatly improved the conidiation; and the loss of the two genes caused a tremendous reduction of the conidiation. These results suggested that the DCLs were important for the normal vegetative growth and conidiation of *C. gloeosporioides*. Furthermore, only the double mutant showed severe impair on the normal growth and conidiation, suggesting functional redundancy between DCL1 and DCL2.

Anthracnose of rubber tree mainly occurred at the tender and wounded leaves (Cai et al., 2013). In the present study, the pathogenicity of the *C. gloeosporioides* was accessed by inoculating the conidia of the WT and mutants to the detached “light green” leaves without wound. The results showed that ΔDcl1ΔDcl2 caused no lesion on the leaves of *Hevea brasiliensis*, indicating the complete loss of pathogenicity; whereas both ΔDcl1 and ΔDcl2 showed similar pathogenicity as WT. The conidia viability and successful penetration are critical steps for many fungal pathogens to infect host plants. Our results showed that the germination rate of conidia of ΔDcl1ΔDcl2 mutant was nearly same as the WT when cultured on the surface of cellophane with sufficient nutrient (Figures 4B,C). When inoculated on onion epidermis, conidia of the WT could successfully penetrate into the onion epidermis after 12 h and formed complex networks in the onion cells after 20 h; but those of ΔDcl1ΔDcl2 mutant did not geminate at all after 12 h and only grow on the surface instead of penetrating into the onion cells after 20 h (Figure 4A). These results suggest that loss of penetration ability was the main cause for the decreased pathogenicity of the ΔDcl1ΔDcl2 mutant.

Oxidative burst is one of earliest events in the plant hypersensitive response to the pathogen attack. In the present study, DAB staining was used to analyze the hydrogen peroxide accumulation. The results showed that the mechanical damage could induce a slightly peroxide accumulation in CK. When inoculated with the WT strain of *C. gloeosporioides*, there was a significantly increase of hydrogen peroxide all over the leaves; but the leaves inoculated with ΔDcl1ΔDcl2 did not induce the oxidative burst, indicating that the interaction between the host and the pathogen was also impaired in ΔDcl1ΔDcl2.

Small RNAs were proved to play multiple functions in living cells, including the regulation of interaction between plant and pathogens (Padmanabhan et al., 2009; Li et al., 2013; Pumplin and Voinnet, 2013; Ouyang et al., 2014). In order to identify the potential targets of DCLs in *C. gloeosporioides* cells, proteome profiles of the wild type and ΔDcl1ΔDcl2 mutant were analyzed. Using the 2D gel electrophoresis, a total of 72 proteins spots were identified to be down-regulated in the ΔDcl1ΔDcl2 mutant. First, 6 spots representing 2 proteins related to cell cycle and 12 spots represented 9 proteins related to proteins synthesis were down regulated in the double mutant. Protein biosynthesis are the basis for normal cell growth and cell division. The decrease in expression of these two categories of proteins caused the significant impair on the hyphae growth and cell division, which lead to the depression of vegetative growth and conidiation processed (Figure 2). Chaperones are required for macromolecules to fold correctly and perform their normal biological functions. In the study, we identified 5 proteins related to polypeptide stability and folding, indicating the complicated influences of DCLs on the protein synthesis. Second, abundance of 6 proteins involved in the transport and 3 cell structure proteins were significantly reduced. Among them, the Rab GTPase, Ras GTPases and clathrin were all reported to be involved in the vesicle transport (Salminen and Novick, 1987; Dumas et al., 2001; Gall et al., 2002); protein disulfide-isomerase and alpha-mannosidase are involved in the modification of secreted proteins; actin also participate in the substance transport, in addition to its function in cytoskeleton formation (Gottlieb et al., 1993). According to the previous report, impair of the vesicle transport could also induced the decrease of the cell growth, development, and the pathogenicity of pathogens (Zhang et al., 2014), which were in accordance with our results (Figures 2, 3). It is well known that, in order to facilitate the penetration process, fungal pathogens could secret abundance of hydrolytic enzymes to degrade the cell wall or defense proteins of the host plant. Here we found that 4 hydrolytic enzymes, including aspergillopepsin-2, endochitinase, aspartic endopeptidase and proteasome endopeptidase, were decreased in the double mutant, which may impair the penetration ability of the pathogen (Figure 4). 5 proteins (8 spots) related to signal transduction were also down regulated in the double mutant, indicating that DCLs also function as signal regulators. Moreover, 9 identified proteins are related to metabolisms were also down-regulated, including tricarboxylic acid cycle, alcohol metabolism, fatty-acid and isoprenoid metabolism, superoxide metabolism and antibiotic response proteins, indicating the diverse functions of the DCLs. Taking together, the proteomics assays revealed that DCLs regulate vegetative growth and conidiation by delaying the cell cycle, repress the protein synthesis and even the disturbance of the cell skeleton. Fungal pathogenicity is up to many biological processes, including the attachment of conidia to host plant surface, conidia germination, hyphal penetration, and overcoming of plant immunity. In the present study, a series of proteins directly related to or involved in the fungal pathogenicity were significantly down regulated, which may be the main cause of the loss of pathogenicity of the mutant. It has been reported that DCLs could affect the transcript levels of the target genes via generation of small RNAs (Li et al., 2013; Ouyang et al., 2014). In the present study, the quantitative RT-PCR assays showed that the transcript levels of 16 selected genes were significantly decreased, and results were in consistent with the variation in abundance of relative protein, suggesting that the DCLs regulated protein abundance via affecting the transcript levels of relative genes.

To summarize, DCLs regulate the growth, conidiation and pathogenicity by affecting the expression of a series of functional proteins in *C. gloeosporioides*.

## Author contributions

QW and BA conceived and designed this study. QW, XH, and YG performed the experiments. BA and HL wrote the manuscript. CH provided critical advice. All authors approved the final manuscript to be published.

### Conflict of interest statement

The authors declare that the research was conducted in the absence of any commercial or financial relationships that could be construed as a potential conflict of interest.
