# Supplementary material for: Dicer-like Proteins Regulate the Growth, Conidiation, and Pathogenicity of Colletotrichum gloeosporioides from Hevea brasiliensis
Source: Front Microbiol. 2018 Jan 4;8:2621. doi: 10.3389/fmicb.2017.02621 (PMC5777394; doi:10.3389/fmicb.2017.02621)
Supplement: Supplementary file 1 [file Table2.DOCX]

Table S1 PCR primers used in this study.

|  | Primer | Sequence (5′→3′) | Application |
| --- | --- | --- | --- |
| 1 | Dcl1-5F | ggaattcAAGCAGGATGTTGATGG | dcl1 deletion |
| 2 | Dcl1-5R | cccaagcttATTGGTGTCTCGGAGTAG | dcl1 deletion |
| 3 | Dcl1-3F | gctctagaAGTGCAGGGATGGAGGGG | dcl1 deletion |
| 4 | Dcl1-3R | ggaattcAGCGGAATGGAATTGGAAGG | dcl1 deletion |
| 5 | Dcl2-5F | ggaattcCCCTGGTCCTTCTGCCTAC | dcl2 deletion |
| 6 | Dcl2-5R | cccaagcttTACCACGGTGCCACGGTG | dcl2 deletion |
| 7 | Dcl2-3F | gctctagaTGTTTGGCGCCATCTGACG | dcl2 deletion |
| 8 | Dcl2-3R | ggaattcGCGAGTTTGCCGAGTA | dcl2 deletion |
| 9 | Dcl1-SF | GGGATGGACGACTTGGACTCAGCA | ∆Dcl1 diagnosis |
| 10 | PCB-SR | AACAACGAGGAGCAGGGAATGGTG | ∆Dcl1 diagnosis |
| 11 | PCB-SF | CCAGGCTTTACACTTTATGCTTCC | ∆Dcl1 diagnosis |
| 12 | Dcl1-SR | ACATGACGTATTACGCCACTACCA | ∆Dcl1 diagnosis |
| 13 | Dcl2-SF | GCGAGTGGATTGCGAGTGCAAATT | ∆Dcl2 diagnosis |
| 14 | PCB-SR | AACAACGAGGAGCAGGGAATGGTG | ∆Dcl2 diagnosis |
| 15 | HPH-SF | ACAGCGGTCATTGACTGGAGCGA | ∆Dcl2 diagnosis |
| 16 | Dcl2-SR | CTCGCCATTCTCAGTTGTCTACCA | ∆Dcl2 diagnosis |
| 17 | Dcl1-F | ATGCTCGTCAAAAAGGAAGC | single conidia isolation |
| 18 | Dcl1-R | TACTGCAGTGCCATGATTGA | single conidia isolation |
| 19 | Dcl2-F | ATGGCTGACTATTCCCAATC | single conidia isolation |
| 20 | Dcl2-R | GTCAGCATCGTCGTTCTCGA | single conidia isolation |
| 21 | Dcl1-Com-F | gctctagaGGATGCGAAGACTGATT | ∆Dcl1 complementation |
| 22 | Dcl1-Com-R | cgggatccTTATACTGCAGTGCCATG | ∆Dcl1 complementation |
| 23 | Dcl2-Com-F | tccccgcggGTTCTTGGAGCCTGGAC | ∆Dcl2 complementation |
| 24 | Dcl2-Com-R | gctctagaCTAGTCAGCATCGTCGTT | ∆Dcl2 complementation |
| 25 | Qtubulin1-F | TCCCGAACAATGTGCAGACA | qPCR assays |
| 26 | Qtubulin1-R | AGAACGCCTTTCTGCGAAAC | qPCR assays |
| 27 | Qspot1-F | CGTCAACAACCTCGGTACCA | qPCR assays |
| 28 | Qspot1-R | TCGTCATCGTTGTGCTTGGA | qPCR assays |
| 29 | Qspot5-F | CAGGCCGATATGAAGCACTTC | qPCR assays |
| 30 | Qspot5-R | AGGACCATGGCGGAGATCT | qPCR assays |
| 31 | Qspot11-F | TCGACAATGGTTCGGGTATGT | qPCR assays |
| 32 | Qspot11-R | TGTGCCTCATCACCGACGTA | qPCR assays |
| 33 | Qspot12-F | ATCACCTCCCTCCAGCACAA | qPCR assays |
| 34 | Qspot12-R | GAGCTGGAGTCTTCGCTGAAG | qPCR assays |
| 35 | Qspot14-F | ACCTTCGACGCTTGGTACGA | qPCR assays |
| 36 | Qspot14-R | GTCTTGCCGGTGGTCAGGTT | qPCR assays |
| 37 | Qspot16-F | AGGAGGACTTGGCGTTTGC | qPCR assays |
| 38 | Qspot16-R | TCGGAACACATCCAAATCGA | qPCR assays |
| 39 | Qspot17-F | CATTGGCGACTTCGAATTCA | qPCR assays |
| 40 | Qspot17-R | GACGACTGGCAATCCCAGAT | qPCR assays |
| 41 | Qspot20-F | ACGAGGAGATGGTCGAGAACA | qPCR assays |
| 42 | Qspot20-R | GCTCGATGGAGGTGACGATT | qPCR assays |
| 43 | Qspot26-F | TTTTGGTGCAAATGCCAATC | qPCR assays |
| 44 | Qspot26-R | ATGTGGTGCCAGCGTTCAT | qPCR assays |
| 45 | Qspot29-F | TGCGCCTTTGGGATATCAAG | qPCR assays |
| 46 | Qspot29-R | TGATGTCGACGACGATGATGT | qPCR assays |
| 47 | Qspot32-F | CACCACGTTCCAGCAACAGA | qPCR assays |
| 48 | Qspot32-R | ATGAACTTGCAGCCGAAAGAC | qPCR assays |
| 49 | Qspot42-F | TCATCGCAGAGGAGTCCACAT | qPCR assays |
| 50 | Qspot42-R | TTCTTCGACGTAGGCGCTGTA | qPCR assays |
| 51 | Qspot45-F | CTGCCTTTGAGGCCATGAA | qPCR assays |
| 52 | Qspot45-R | AGGCCCTTGGTCTCGAAGA | qPCR assays |
| 53 | Qspot50-F | CCACACCAAGGACCTCAACAC | qPCR assays |
| 54 | Qspot50-R | CTGCCGATTCCTGACTCTTTG | qPCR assays |
| 55 | Qspot56-F | CCCCTCTGAGGTTCTCGACAT | qPCR assays |
| 56 | Qspot56-R | GCCGTTGAAGAAGGAGTGGAT | qPCR assays |
| 57 | Qspot71-F | CCCGTGCTGGTATCCTGAA | qPCR assays |
| 58 | Qspot71-R | CTTGACAGCACGCTGGGTAA | qPCR assays |


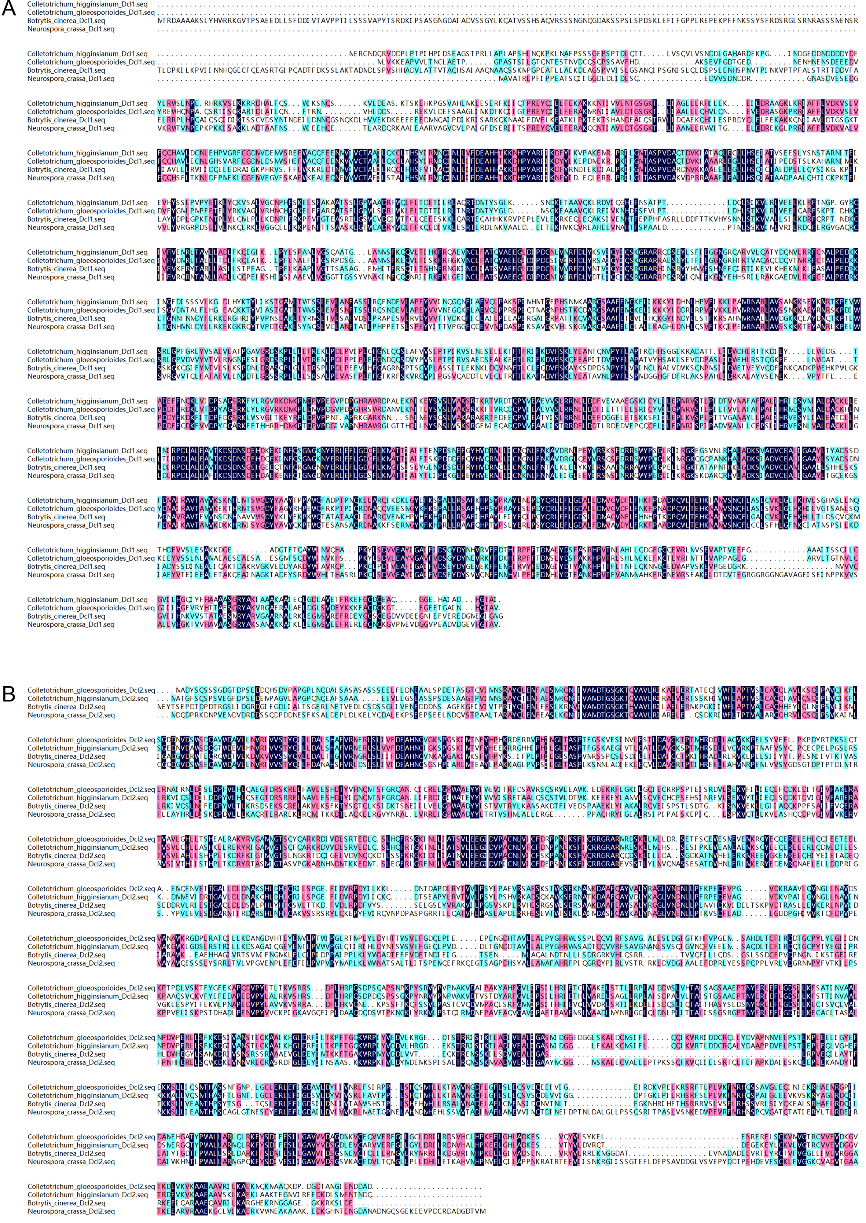


Figure S1 Alignment of Dicer like protein sequences from *Colletotrichum gloeosporioides*, *Colletotrichum higginsianum*, *Botrytis cinerea* and *Neurospora crassa*. **(A)** Alignment of DCL1 proteins. **(B)** Alignment of DCL2 proteins.


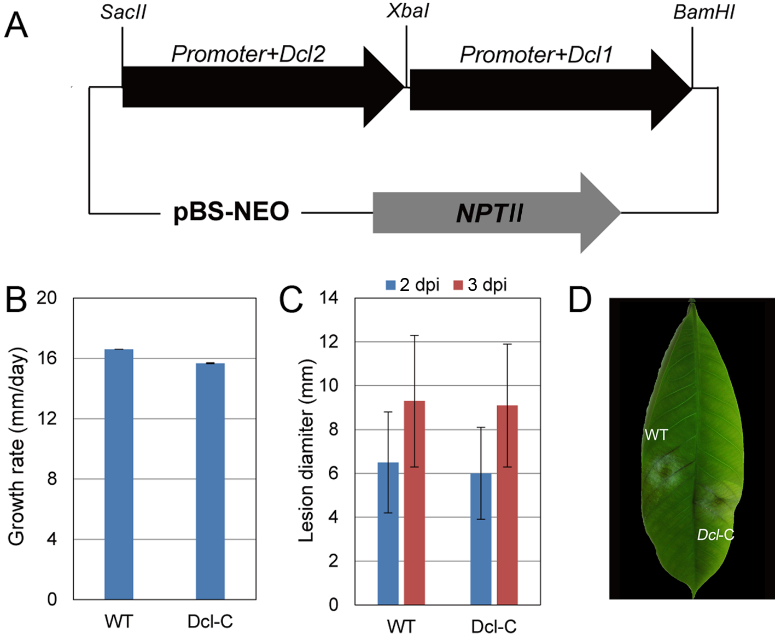


Figure S2 The complementation strategy of the ΔDcl1ΔDcl2 mutant and the phenomenon assays of the complementary mutant (*Dcl-C*). (**A**) The complementation strategy of the ΔDcl1ΔDcl2 mutant. **(B)** Growth rate of WT and the *Dcl-C* mutant strains on CM for 5 days. **(C)** Mean lesion diameters after 2 and 3 dpi. Bars represent standard deviations (SD). **(D)** Disease symptoms of rubber tree leaves at 3 day post inoculation.
